# Ying‐Huang Oral Liquid Ameliorates Oxidative Stress in the Liver of Heat‐Stressed Broilers via KEAP1‐NRF2 and PINK1/Parkin Pathway

**DOI:** 10.1002/fsn3.71660

**Published:** 2026-03-15

**Authors:** Zi‐Hao Li, Jia‐Ci Cai, Yang Yang, Hui‐Lin Li, You Li, Shun Tian, Yong‐Ming He, Lu‐Ping Tang

**Affiliations:** ^1^ School of Animal Science and Technology Foshan University Foshan China

**Keywords:** heat stress, liver injury, network pharmacology, Nrf2, Sirt1, Yinhuang oral liquid

## Abstract

Heat stress (HS) impairs liver function and negatively affects the growth performance of poultry. This study evaluates the protective effects and underlying mechanisms of Yinhuang Oral Liquid (YOL) in alleviating liver injury in heat‐stressed broilers, highlighting its potential application in mitigating HS‐related liver damage. Broilers were randomly allocated into six groups: Control (23°C ± 2°C), Model (HS, 35°C ± 2°C for 8 h/day for 7 consecutive days), Positive (HS + Vitamin C, 60 mg/kg), YOL‐H (HS + YOL, 10 g/kg), YOL‐M (HS + YOL, 5 g/kg), and YOL‐L (HS + YOL, 2.5 g/kg). Vitamin C and YOL were administered intragastrically 1 h after HS exposure for 7 days. HS impaired growth performance, as evidenced by decreased final body weight, average daily feed intake (ADFI), and average daily gain (ADG), along with an increased feed conversion ratio (FCR). YOL supplementation increased ADG compared with the model group. Histopathological analysis showed severe inflammatory cell infiltration around the hepatic central vein under heat stress, which was markedly attenuated by vitamin C and YOL. YOL activated the hepatic NRF2–KEAP1 signaling pathway by increasing the p‐P62/P62 ratio, increasing NRF2 and its downstream antioxidant proteins HO‐1 and NQO1, and downregulating KEAP1 expression. Network pharmacology analysis identified Sirt1 as a crucial bridging target linking YOL to heat stress. Molecular docking further demonstrated that the top four compounds—baicalin, wogonoside, oroxylin A‐7‐O‐glucuronide, and acacetin‐exhibited high binding affinity with the Sirt1 receptor. YOL significantly upregulated Sirt1, GPX4, and SLC7A11 protein levels and restored mitochondrial cristae integrity while promoting autolysosome formation. It also restored HS‐induced suppression of PINK1 and Parkin. In conclusion, YOL effectively mitigates HS‐induced oxidative stress in broilers by activating Sirt1, which coordinately enhances the NRF2–KEAP1 and PINK1/Parkin signaling pathways.

## Graphical abstract

Protect liver health and enhance growth performance: ensure safer, higher‐quality meat, and improve economic efficiency in poultry production. Heat stress induces liver injury through oxidative stress, ferroptosis, and mitochondrial dysfunction. Yinhuang oral liquid (YOL) mitigates these effects via multi‐target mechanisms, including activation of the KEAP1–NRF2 antioxidant pathway, inhibition of ferroptosis, and restoration of mitochondrial integrity through the PINK1/Parkin pathway. YOL administration increases broiler resilience to heat stress, reducing susceptibility to infectious diseases and minimizing the need for veterinary interventions.

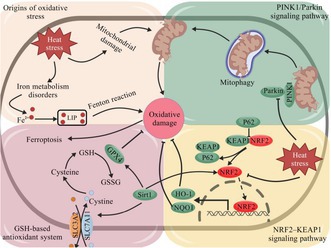

## 1 Introduction

The global poultry industry is currently confronted with multiple challenges, including climate change‐induced temperature fluctuations, rising demand for poultry products, and the ongoing emergence and spread of infectious diseases (Ali Khan et al. 2025). These challenges are closely associated with heat stress. Unlike mammals, poultry are covered with dense feathers and lack functional sweat glands, severely limiting their capacity for effective thermoregulation under high ambient temperatures (Mota‐Rojas et al. 2021). Modern intensive production systems—characterized by high stocking densities, rapid genetic selection for accelerated growth, and elevated metabolic heat production—further exacerbate the susceptibility of poultry to heat stress (Averós and Estevez 2018). Heat stress significantly impairs growth performance, feed efficiency, reproductive capacity, and survivability, posing a significant threat to the stability of global poultry‐derived protein supplies (Ahmad et al. 2022). Moreover, heat stress‐induced immunosuppression increases susceptibility to disease, raising further concerns for food safety, as affected flocks are prone to pathogen contamination, mycotoxin accumulation, and compromised carcass quality (Zmrhal et al. 2023).

To mitigate the adverse effects of heat stress, various preventive and control strategies have been developed, primarily focusing on management, genetic, and nutrition (Onagbesan et al. 2023). Management strategies focus on reducing environmental heat load through enhanced ventilation, cooling systems, and housing modifications; however, these interventions can substantially increase production costs and may not be feasible in resource‐limited regions (Goel 2021). Genetic strategies, including the selection of heat‐resistant genotypes, early‐life thermal conditioning, and embryonic manipulation, offer potential for long‐term adaptation to heat stress (Fathi et al. 2022; Fernandes et al. 2023). However, trade‐offs remain, such as negative correlations between heat tolerance and economically important traits. Embryonic manipulation is limited by low heritability in certain species and carries the risk of unintended genetic disruptions that may increase susceptibility to other diseases (Murakami 2024). Nutritional strategies, including feed restriction, supplementation with vitamins, minerals, electrolytes, as well as the use of herbal additives, represent flexible and widely applied interventions (Wasti et al. 2020). While feed restriction can reduce metabolic heat production, it may also extend growth cycles. Micronutrient supplementation can alleviate oxidative stress and help maintain acid–base balance, thereby improving performance under heat stress exposures (Zhou et al. 2025). Among these interventions, herbal additives are particularly promising due to their low cost, safety, lack of residues, and diverse biological activities. By modulating immune responses, enhancing antioxidant capacity, and improving stress tolerance through multiple targets and biochemical pathways, herbals are increasingly recognized as valuable candidates for alleviating heat stress–induced physiological dysfunction in poultry (Baird and Yamamoto 2020).

Accumulating evidence indicates that heat stress induces pronounced hepatic injury in poultry, characterized by excessive oxidative stress and inflammation, which ultimately disrupts metabolic homeostasis and reduces growth and production performance (Ma et al. 2025). Our previous research further demonstrated a direct link between heat stress‐induced liver damage and impaired performance in poultry (Tang et al. 2022), highlighting the liver as a key target organ in thermal stress responses. These findings suggest that targeting hepatic dysfunction with Traditional Chinese Medicine (TCM) may provide a promising preventive and therapeutic strategy.

Yinzhi jiedu Granule, listed in the second volume of the *Compilation of National Standards for Veterinary Drugs*, is primarily composed of *Artemisia capillaris*, *Gardenia jasminoides*, *Scutellaria baicalensis*, *Polygonum cuspidatum*, and *Uncaria rhynchophylla*, and is among the few officially approved hepatoprotective formulations (Veterinary Drug Evaluation Center of the Ministry of Agriculture 2010). Guided by traditional principles of heat clearance, detoxification, liver regulation, and antispasmodic effects, this formulation has been widely used to support hepatic function in livestock and poultry (Liu et al. 2021). In a previous study, Yinzhi jiedu granule was separated with a dismantling study, and obtained the formula of *Artemisiae scopariae* Herba: *Scutellaria baicalensis* Georgi (Volume 2:1), which was designated as Yinhuang Oral Liquid (YOL). The preparation process was standardized, quality control criteria were established, and the safety margin was determined (Li et al. 2020). Subsequent studies demonstrated that YOL provides protective effects against drug‐induced liver injury (DILI) (He et al. 2022). However, the precise molecular mechanisms underlying heat stress‐induced liver injury in poultry remain incompletely elucidated. Therefore, elucidating the biological mechanisms underlying heat stress‐induced hepatic dysfunction and identifying effective pharmacological interventions are imperative. In this study, YOL was evaluated as a potential hepatoprotective agent against heat stress, with the objectives of characterizing heat stress‐induced liver injury and elucidating the molecular mechanisms underlying its protective effects.

## 2 Materials and Methods

### 2.1 Animals and Experimental Design

A total of 120 two‐week‐old SPF healthy male Ma chickens were provided by the SPF Laboratory Animal Center of Wen Xinxing Dahua Nong Poultry and Egg Co. LTD (Guangdong, China). All broilers underwent a 7‐day acclimatization period in an environmentally controlled room maintained at 23°C ± 2°C with a 12‐h light/dark cycle. Following acclimatization, the broilers were randomly assigned by body weight into six groups (*n* = 20 per group): Control (maintained at 23°C ± 2°C), Model (Heat Stress, HS; exposed to 35°C ± 2°C for 8 h/day for 7 days), Positive (HS + vitamin C), and three Yinghuang Oral Liquid (YOL) treatment groups (YOL‐H, 10 g/kg; YOL‐M, 5 g/kg; YOL‐L, 2.5 g/kg). Broilers in all HS and treatment groups were subjected to the same daily 8‐h (HS from 8:00 am to 16:00 pm) heat stress protocol, and the rest of the time was consistent with that of control group. One hour after HS initiation, chickens received intragastric administration of either vitamin C (60 mg/kg/d) or YOL at the respective doses. Throughout the entire experiment, all broilers had ad libitum access to feed and water. The doses of were determined based on a combination of our previous mouse studies, and the dosage conversion between the specific species and experimental animals (He et al. 2022).

### 2.2 Sample Collection

The body weight was recorded at the start of the experiment (3 weeks of age) and at the end of the experiment (4 weeks of age). Daily feed intake and feed residues were recorded to calculate average daily feed intake (ADFI), average daily gain (ADG), and feed conversion ratio (FCR). At the end of the trial, broilers were euthanized in accordance with the Laboratory animal—Guidelines for euthanasia (GB/T 39760‐2021), and the liver tissues were immediately collected. The left liver lobe was divided into two portions and fixed in 4% paraformaldehyde and 2.5% glutaraldehyde for histopathological and ultrastructural analyses, respectively. The right liver lobe was snap‐frozen and stored at −80°C for subsequent biochemical and molecular analyses.

### 2.3 Iron Content in Liver Tissue

Liver tissues were homogenized in 0.9% normal saline and centrifuged at 2500 g/min for 10 min at 4°C. The resulting supernatants were collected, and iron concentration was determined via the colorimetric method provided in the kit, with absorbance measured at 550 nm. Each sample was analyzed in triplicate. Hepatic iron concentrations were measured using a commercial iron assay kit obtained from Nanjing Jiancheng Bioengineering Institute (Nanjing, China). The specific test operation method was carried out following the manufacturer's instructions.

### 2.4 Glutathione (GSH) Content in Liver Tissue

The hepatic glutathione (GSH) content was determined using a reduced glutathione assay kit (No. A006‐1‐1) purchased from Nanjing Jiancheng Bioengineering Institute. Approximately 0.1 g of fresh liver tissue from broilers in each group was homogenized with 0.9 mL of physiological saline using a tissue homogenizer to prepare a 10% (w/v) liver homogenate. The GSH level in the homogenate was then quantified according to the manufacturer's instructions.

### 2.5 Pathological Analysis of Liver

Liver tissues were fixed in 4% paraformaldehyde for 24 h, dehydrated through a graded ethanol series, cleared in xylene, embedded in paraffin, and sectioned at 4 μm thickness. The sections were stained with hematoxylin and eosin (H&E) using standard procedures. Histopathological features and morphological alterations were observed under a light microscope (Mshot, Guangzhou, China).

### 2.6 Transmission Electron Microscopy of Liver Tissue

The fresh liver samples were immediately cut into 1–2 mm^3^ pieces and immersed in 2.5% glutaraldehyde for primary fixation. The tissues were fixed at room temperature in the dark for 2 h, and subsequently stored at 4°C. Following gradient ethanol dehydration, samples were embedded in epoxy resin, ultrathin‐sectioned, and sequentially stained with uranyl acetate and lead citrate. Mitochondria morphology and ultrastructural alterations were examined using a transmission electron microscope (JEOL Ltd., Japan).

### 2.7 Network Pharmacology and Molecular Docking Analysis

The active components and their relative abundances in Yinghuang Oral Liquid were analyzed using HPLC‐MS. Potential targets of these components were collected from the SwissTargetPrediction and TCMIP databases. Heat stress‐related targets were retrieved from the OMIM, TTD, GeneCards, and DisGeNET databases. Ferroptosis‐related targets were obtained from the FerrDB database.

The common targets were used to construct a protein–protein interaction (PPI) network, from which core targets were screened based on degree and betweenness centrality values. An “Ingredient‐Target” network was then established. Key active components were prioritized using an evaluation score derived from topological parameters. (Evaluation Score = (Normalized Betweenness × 0.4 + Normalized Degree × 0.4 + Normalized Closeness × 0.2) × 100 × Relative Abundance).

The screened high‐priority active components were then subjected to molecular docking with the identified core targets. Docking calculations were performed using AutoDock Vina, and the results were visualized using PyMOL software.

### 2.8 Western Blot Analysis

Toal protein was extracted from liver tissues using RIPA lysis buffer supplemented with phosphatase inhibitors on ice. After centrifuged at 13000 r/min for 10 min at 4°C, the protein concentration of the supernatant was determined using an Enhanced BCA Protein Assay Kit (Cat. #P0010, Beyotime, China). Equal amounts of protein were separated by SDS‐PAGE using gels with appropriate acrylamide concentrations according to molecular weight and subsequently transferred onto polyvinylidene difluoride (PVDF) membranes. Membranes were blocked with 5% skin milk (Cat. #9999, CST, USA) for 1 ~ 3 h at room temperature, followed by incubation with the respective primary antibodies at 4°C overnight. After washing 3 times with TBST, memberanes were incubated with horseradish peroxidase (HRP)‐conjugated secondary antibodies for 1 h at room temperature. Protein bands were visualized using an enhanced chemiluminescence (ECL) detection kit (Cat. #170‐5051, Bio‐Rad, USA) and imaged using a chemiluminescence image system (Tanon‐5200SF, Shanghai, China). Band intensities were quantified using ImageJ software.

Primary antibodies targeting P62 (Cat. #39749), phospho‐P62 (Cat. #16177), KEAP1 (Cat. #4678), HO‐1 (Cat. #86806), NQO1 (Cat. #8089), Sirt1 (Cat. #2011), SLC7A11 (Cat. #12994), GPX4 (Cat. #62262), and GAPDH (Cat. #5174) were purchased from Cell Signaling Technology (Danvers, MA, USA). The NRF2 antibody (Cat. #WL02135) was obtained from Wanlei Bio (Shenyang, China). HRP‐conjugated rabbit IgG (Cat. #SA00001‐2) and mouse IgG (Cat. #SA00001‐1) secondary antibodies were purchased from Proteintech (Wuhan, China).

### 2.9 Statistical Analysis

Data are presented as means ± standard deviation (SD). All statistical analyses were performed using GraphPad Prism version 8.0.2 (GraphPad Software Inc., San Diego, CA, USA). Analysis strictly adhered to the traditional parametric statistical framework and, where necessary, incorporated robust extensions to address potential deviations from the underlying assumptions of the data. The normality of data distribution was assessed using the Shapiro–Wilk test, while homogeneity of variance was evaluated by the *F*‐test or Brown‐Forsythe test, as appropriate. For data meeting the parametric assumptions, one‐way ANOVA was employed to test overall differences in means between the multiple treatment groups and the model control group.

For comparisons between two groups, an unpaired Student's *t*‐test was used when variances were equal, and Welch's *t*‐test was applied if variances were unequal. For multiple‐group comparisons with normally distributed data, Dunnett's test was used when variances were equal, and Dunnett's T3 test was employed for unequal variances. Nonparametric tests were used for data that did not conform to normal distribution. A *p*‐value < 0.05 was considered statistically significant.

## 3 Results

### 3.1 YOL Improved the Growth Performance of Broilers Exposed to Heat Stress

Growth performance data are presented in Table 1. Final body weight, ADFI and ADG were significantly reduced in the model group compared with the control group (all *p* < 0.05), while FCR was markedly elevated (*p* < 0.05). Administration of YOL‐L and vitamin C effectively reduced FCR relative to the model group (both *p* < 0.05). No mortality was observed in any group thgoughout the experimental period.

**TABLE 1:** The growth performance of broilers.

Group	Initial body weight (g)	Final body weight (g)	ADFI (g)	ADG (g)	FCR
Control	195.80 ± 24.15	289.50 ± 36.89	33.63 ± 11.66	13.39 ± 1.90	2.55 ± 0.34
Model	194.00 ± 16.52	243.80 ± 21.48^a^	26.70 ± 8.52^a^	7.11 ± 0.96^a^	3.82 ± 0.55^a^
Positive	195.60 ± 24.29	258.60 ± 34.03	29.32 ± 9.25	9.00 ± 1.55^b^	3.34 ± 0.16
YOL‐L	193.90 ± 26.45	255.40 ± 36.32	28.63 ± 6.79	9.61 ± 1.42^b^	3.50 ± 0.17
YOL‐M	194.60 ± 18.33	245.20 ± 19.05	25.97 ± 5.77	7.66 ± 0.87	3.42 ± 0.37
YOL‐H	196.60 ± 25.67	250.40 ± 28.44	26.99 ± 5.60	8.01 ± 1.58	3.47 ± 0.84

*Note:* The average daily feed intake (ADFI), average daily gain (ADG), and feed conversion ratio (FCR) in broilers.

^a^ *p* < 0.05 vs. Control.

^b^ *p* < 0.05 vs. Model. *n* = 20.

### 3.2 YOL Mitigated the Hepatic Pathological Changes in Broilers Subjected to Heat Stress

**FIGURE 1:**
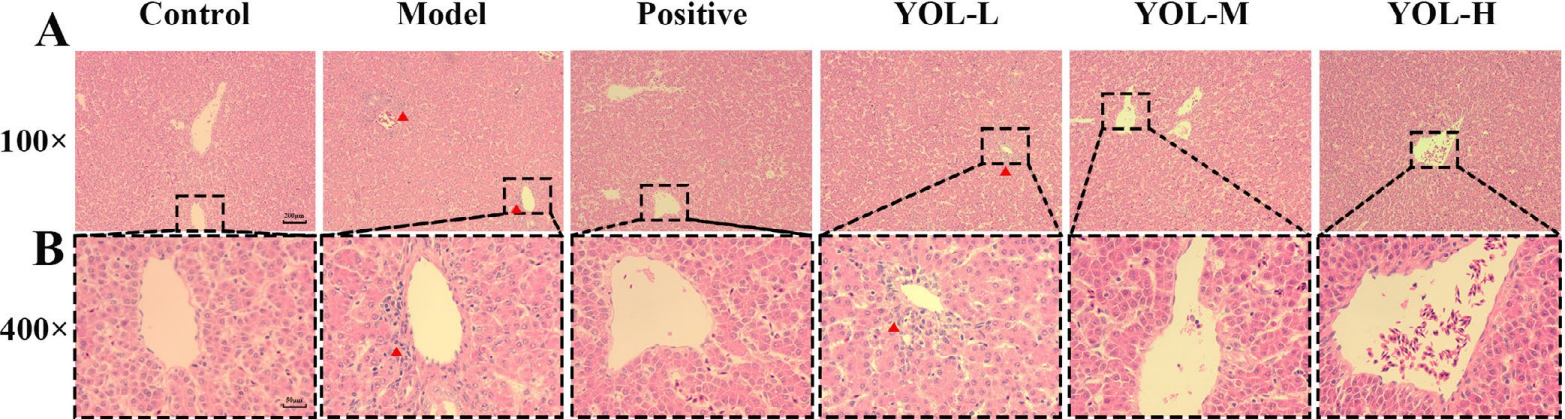
Effect of YOL on hepatic histopathology in heat‐stressed broilers. Liver sections were stained with hematoxylin and eosin (H&E) and examined at ×100 (A) and ×400 (B) magnification. The black box highlights an enlarged view of the central vein, and the red triangle indicates areas of inflammatory cell infiltration.

Histopathological analysis of liver tissues is presented in Figure 1. The control group exhibited intact lobular structure with well‐organized hepatocyte cords and no observable lesions. Heat‐stress broilers displayed pronounced inflammatory cell infiltration around the central vein (red triangles). This infiltration was absent in the VitC, YOL‐M, and YOL‐H groups, whereas mild portal area infiltration persisted in the YOL‐L group. Moreover, medium‐ and high‐dose YOL treatments effectively prevented central vein–associated infiltration, suggesting a dose‐dependent hepatoprotective effect.

### 3.3 YOL Up‐Regulated the NRF2‐KEAP1 Signaling Pathway in Liver of Broilers Subjected to Heat Stress

**FIGURE 2:**
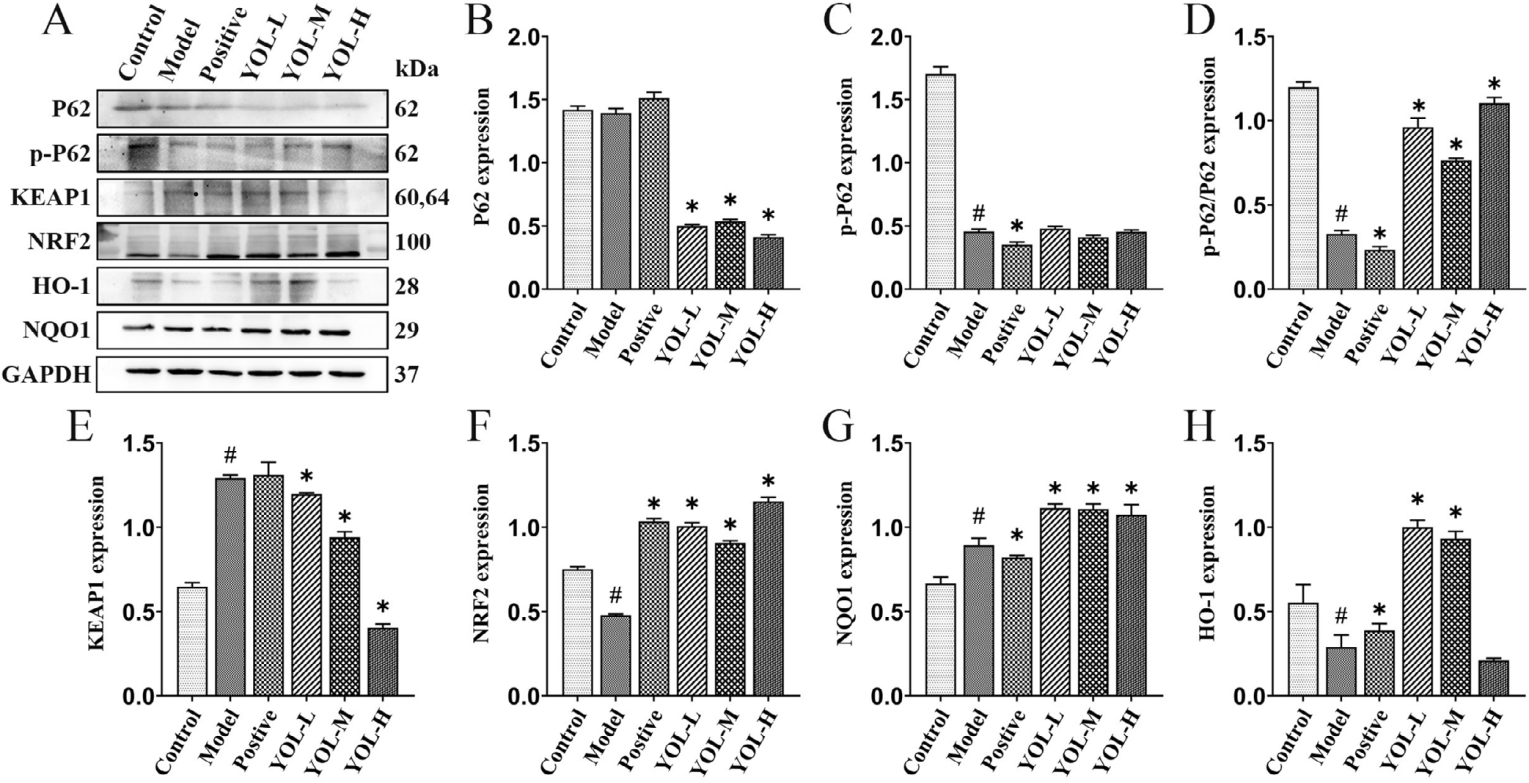
Effect of YOL on the hepatic NRF2–KEAP1 signaling pathway in heat‐stressed broilers. (A) Proteins expressions were measured by western blotting. (B–H) Relative protein abundance were quantified by densitometry and normalized to GAPDH. ^#^ *p* < 0.05 vs. Control group; *p < 0.05 vs. Model group.

As shown in Figure 2A–D, compared with the control group, the model group exhibited no change in total P62 protein expression, but phosphorylated P62 (p‐P62) and the p‐P62/P62 ratio were significantly decreased (*p* < 0.05, *p* < 0.05, respectively). YOL supplementation significantly reduced P62 expression while increasing the p‐P62/P62 ratio (*p* < 0.05). Heat stress also elevated KEAP1 expression (*p* < 0.05) and markedly suppressed NRF2 and its downstream antioxidant protein HO‐1 (*p* < 0.05, *p* < 0.05, respectively, Figure 2E–H). Treatment with vitamin C partially mitigated these alterations. In contrast, YOL administration significantly downregulated KEAP1 and upregulated NRF2, NQO1, and HO‐1 expression (*p* < 0.05 for all). These results indicate that YOL markedly activates the hepatic NRF2–KEAP1 signaling pathway in heat‐stressed broilers.

### 3.4 Core Components and Targets of YOL in Regulating Heat Stress in Broilers

**FIGURE 3:**
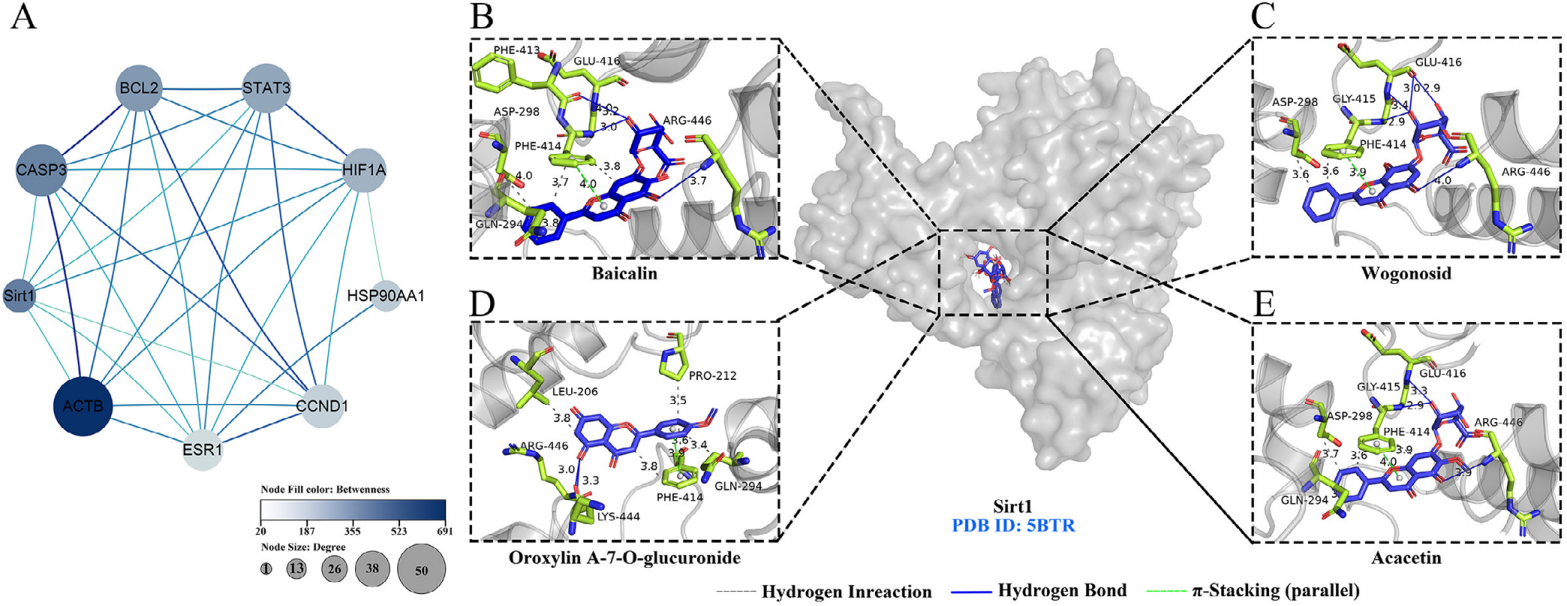
Construction of the PPI network and identification of core targets of YOL and heat stress‐related targets. (A) Protein–protein interaction (PPI) network and core targets screening. The subnetwork contains 9 nodes and 30 edges, where node size represents the degree and color intensity represents betweenness centrality. (B–E) Binding energy profiles of molecular docking between core YOL components (Baicalin, Wogonoside, Oroxylin A‐7‐O‐glucuronide, and Acacetin) and the SIRT1 target.

The intersecting targets were imported into the STRING database to construct a protein–protein interaction (PPI) network, which comprised 70 nodes and 495 edges. To further identify key proteins within the network, a two‐step screening process was performed using Degree and Betweenness values greater than the median as selection criteria, resulting in a refined subnetwork (Figure 3A), consisting of 9 nodes and 30 edges. The deacetylase Sirt1 displayed a high betweenness centrality and a moderate degree, indicating its critical role as a bridging node within the network. Unlike structural hubs such as ACTB, which are directly connected to many nodes, Sirt1 regulates the convergence and flow of multiple key signaling pathways, positioning it strategically for precise regulation of downstream networks. Other core targets, including CASP3, BCL2, and STAT3, demonstrated both hub and bridging functions.

The top 4 ranked compounds, including Baicalin, Wogonoside, Oroxylin A‐7‐O‐glucuronide, and Acacetin, were selected as ligands (Table 2). In the PPI network, Sirt1 was chosen as the receptor due to its moderate degree and high betweenness centrality, reflecting its role in coordinating signaling pathways and enabling precise regulation. The molecular docking results indicated that all four compounds exhibited high binding affinity to the allosteric site of Sirt1, with predicted binding energies below −7 kcal/mol. As shown in Figure 3B–E, each ligand formed at least two hydrogen bonds with Sirt1 in its optimal binding conformation, demonstrating strong and specific interactions between the ligands and the receptor.

**TABLE 2:** Information on the top 10 ingredients in the ingredient‐target network.

Name	Betweenness	Closeness	Degree	Relative abundance (%)	Evaluation score
Baicalin	589.67	0.39	24	36.75	14.17
Wogonoside	590.69	0.40	22	15.07	5.66
Oroxylin A‐7‐O‐glucuronide	590.69	0.40	22	9.01	3.39
Acacetin	3432.33	0.47	48	1.23	1.06
Skullcapflavone II	3034.07	0.46	44	1.00	0.79
Citric acid	295.40	0.32	5	3.24	0.64
Getiacaulein	1468.89	0.36	16	1.57	0.63
Dihydrobaicalin	238.26	0.37	16	1.75	0.50
Chrysin 6‐C‐glucoside 8‐C‐arabinoside	0.00	0.32	1	3.51	0.49
Cryptochlorogenic acid	444.50	0.35	10	1.77	0.46

### 3.5 YOL Enhanced the Expression of Ferroptosis‐Related Proteins in Liver of Broilers Subjected to Heat Stress

**FIGURE 4:**
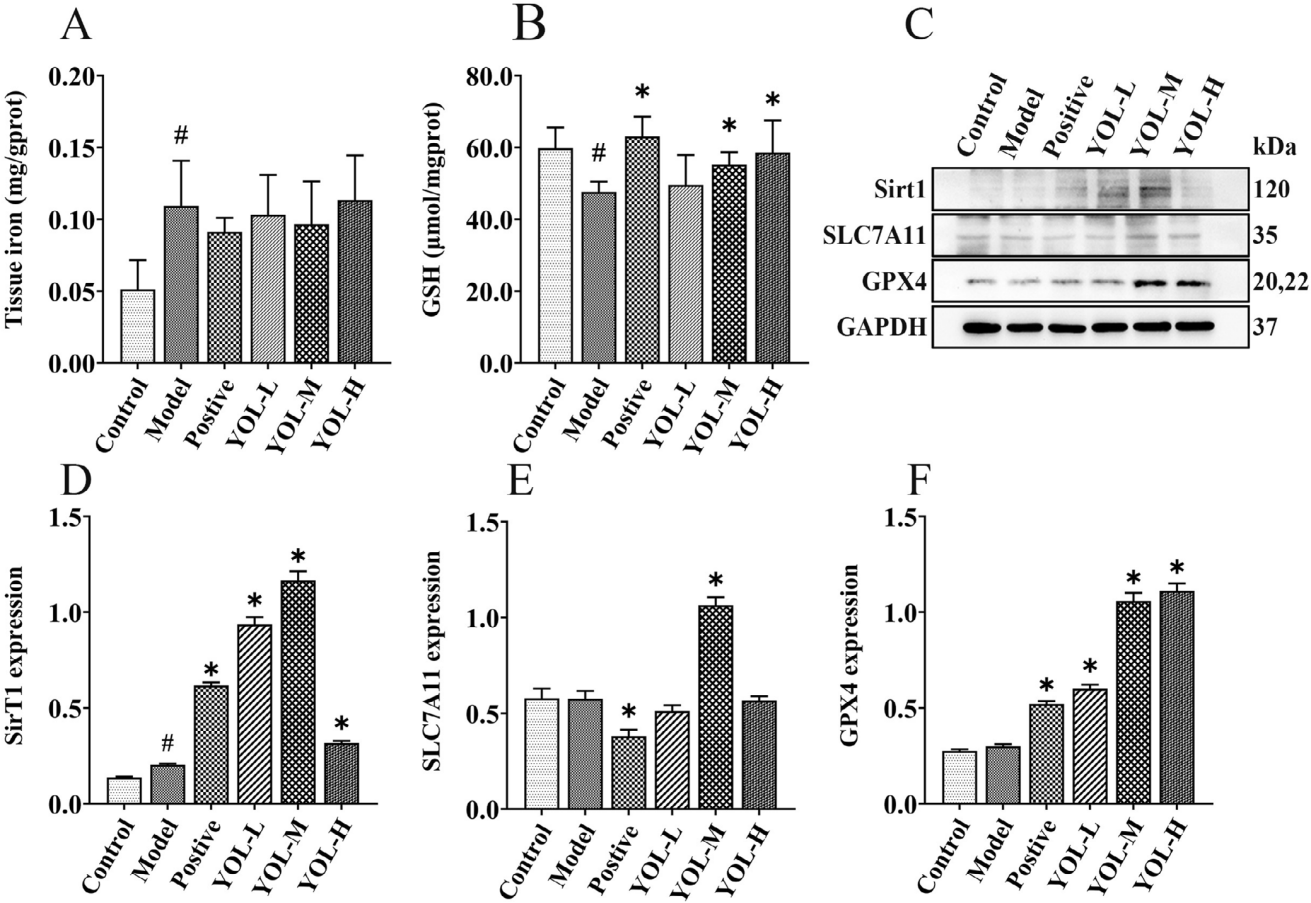
Effect of YOL on hepatic ferroptosis in heat‐stressed broilers. (A) Iron content in liver tissue was measured using an iron assay kit. (B) GSH content in liver was determined using a reduced glutathione assay kit. (C) Protein levels of Sirt1, SLC7A11, and GPX4 were measured by western blotting. (D–F) Relative protein abundance was quantified by densitometry and normalized to GAPDH. ^#^ *p* < 0.05 vs. control group; *p < 0.05 vs. Model group.

Heat stress significantly increased hepatic iron content (*p* < 0.05, Figure 4A), accompanied by an upregulation of Sirt1 protein expression (*p* < 0.05). Compared with the model group, vitamin C significantly enhanced GSH levels, upregulated Sirt1 and GPX4 expression, and downregulated SLC7A11 expression (Figure 4B–F). In contrast, YOL markedly enhanced GSH levels, increased Sirt1, GPX4 and SLC7A11 expression, compared with the model group (*p* < 0.05), suggesting a promotion of ferroptosis‐related protein expression.

### 3.6 YOL Alleviated Mitochondrial Morphology Abnormalities, and Promoted PINK1/Parkin Signaling Pathway in Liver of Broilers Subjected to Heat Stress

**FIGURE 5:**
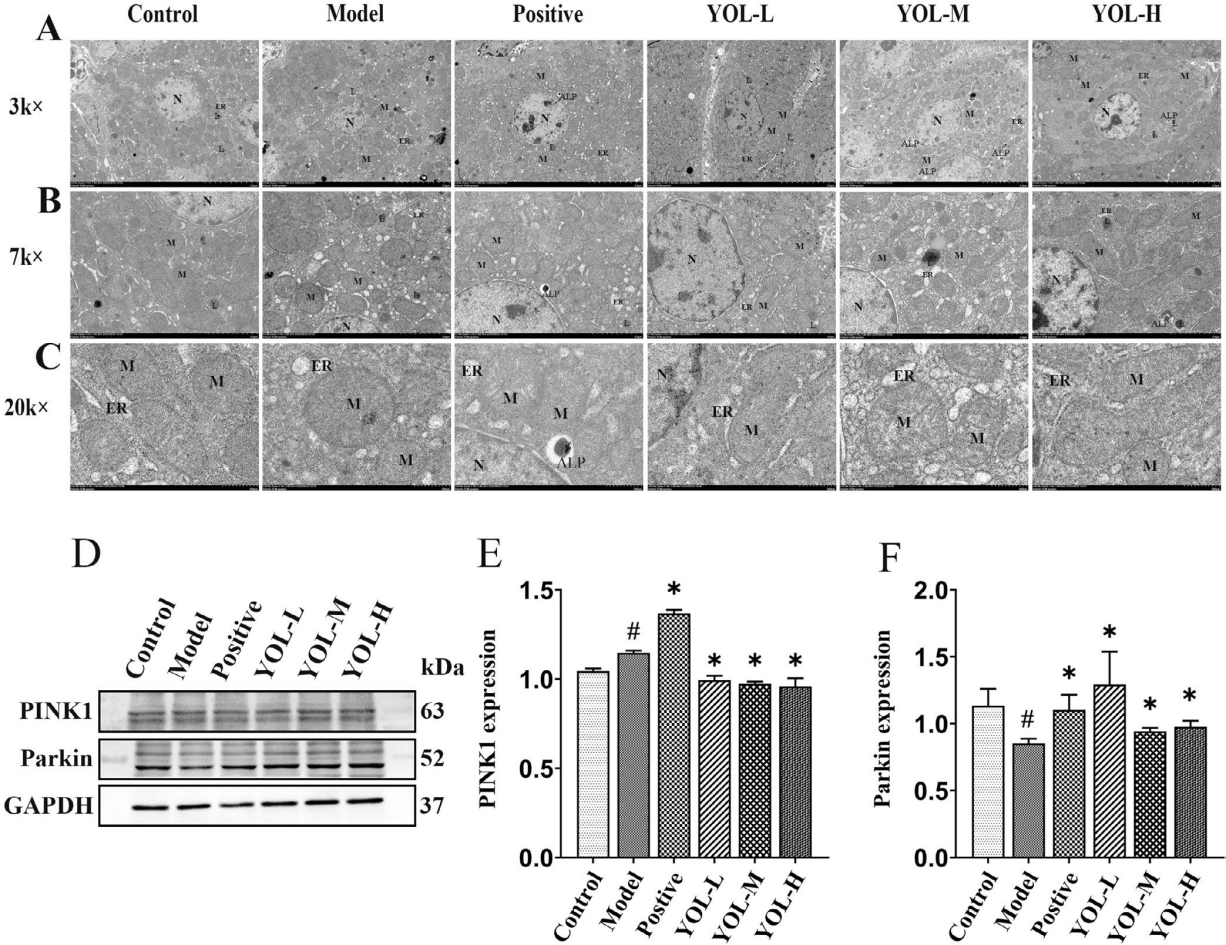
Effect of YOL on hepatic ultrastructure alterations and the PINK1/Parkin signaling pathway in heat‐stressed broilers. (A–C) Ultrastructural alterations in liver were measured by transmission electron microscopy (TEM) at 3 k×, 7 k×, and 20 k × magnification. N, nucleus; M, mitochondria; ER, endoplasmic reticulum; L, lysosomes; ALP, phagolysosomes. (D–F) Protein levels of PINK1 and Parkin were measured by western blotting. ^#^ *p* < 0.05 vs. Control group; *p < 0.05 vs. model group.

In the control group, nuclei exhibited well‐defined envelopes with a typical bilayer structure (Figure 5A–C). Cytoplasmic organelles were clearly delineated, the endoplasmic reticulum displayed distinct tubular morphology, and mitochondria were oval or short‐rod shaped with intact membranes and well‐organized cristae. Exposure to heat stress induced endoplasmic reticulum dilation and a reduction in autolysosome formation. Moreover, mitochondrial ultrastructure was markedly disrupted, characterized by fragmented and largely vanished cristae, partial loss of membrane integrity, and irregular, non–“peanut‐shaped” mitochondrial morphologies. YOL‐L largely restored cristae architecture in most mitochondria. In the vitamin C, YOL‐M and YOL‐H groups, mitochondrial cristae were well‐defined, autophagolysosomes were observed, and endoplasmic reticulum dilation, evident at lower doses, was absent under high‐dose YOL treatment.

Heat stress significantly increased PINK1 and decreased Parkin protein expression compared with the control group (Figure 5D–F *p* < 0.05, *p* < 0.05, respectively). Treatment with vitamin C markedly upregulated both PINK1 and Parkin expression relative to the model group (*p* < 0.05). Vitamin C and YOL restored PINK1 levels and significantly upregulated Parkin expression relative to the model group (*p* < 0.05 for all), indicating that these interventions may partially or fully restore the PINK1/Parkin signaling pathway under heat stress.

## 4 Discussion

Liver health is essential for maintaining optimal performance in poultry (Zaefarian et al. 2019). In this study, heat stress induced inflammatory cell infiltration around the central vein of the liver and inhibited the production performance by reducing ADFI and ADG, while increasing FCR. Supplemention with vitamin C and YOL‐L effectively mitigated growth suppression induced by heat stress.

Heat stress induced liver injury is tightly linked to oxidative stress. The KEAP1‐NRF2 axis functions as a central regulator of cellular antioxidant defenses (Zhou et al. 2025). Under basal conditions, NRF2 is sequestered by KEAP1 in the cytoplasm and targeted for ubiquitin‐dependent degradation. Oxidative stress induces conformational changes in KEAP1, allowing NRF2 to dissociate, translocate to the nucleus, bind antioxidant response elements, and activate downstream antioxidant genes such as HO‐1 and NQO1 (Baird and Yamamoto 2020). In addition, phosphorylated p62 exhibits affinity for KEAP1, relieving KEAP1‐mediated repression of NRF2 (Liu, Pi, and Zhang 2022). Baicalin, an important compound of YOL, ameliorates ethanol‐induced liver oxidative damage via the Nrf2/HO‐1 pathway (Wang et al. 2020). It also attenuates fatty liver disease by suppressing oxidative stress via the p62‐Keap1‐Nrf2 signaling pathway in mice (Liu et al. 2025). In this study, heat stress impaired the KEAP1‐NRF2 antioxidant axis, which was obviously alleviated by YOL. The absence of significant differences in total p62 protein levels among the control, model, and positive drug groups is consistent with the regulatory characteristics of p62. Under heat stress conditions, p62 expression itself remain relatively stable, while its functional state is altered via reduced phosphorylation. Consistently, antioxidant intervention (vitamin C) did not markedly affect total p62 expression or p‐p62/p62 ratio, but partially restored downstream antioxidant responses (restored NRF2 and HO‐1 expression). In contrast, YOL acted at an upstream regulatory level by significantly increased the p‐P62/P62 ratio while reducing total p62 levels, thereby competitively inhibiting KEAP1 and enhancing NRF2 activation. This difference precisely reflects the fundamental distinction in the action targets between YOL and vitamin C: vitamin C direct enhances NRF2 level, while YOL enhances KEAP1‐NRF2 antioxidant axis by activating p62.

Liver is the primary organ for iron storage and particularly susceptible to iron‐driven oxidative injury (Wang et al. 2024). Dysregulation of hepatic iron metabolism can trigger the Fenton reaction, generating excessive free radicals that deplete endogenous antioxidants and induce lipid peroxidation (Zhang et al. 2023). Notably, wogonoside, an active constituent of YOL, has been reported to attenuate liver fibrosis by triggering ferroptosis through the P53/SLC7A11 signaling pathway (Liu, Pi, and Zhang 2022). In this study, heat stress markedly increased hepatic iron levels, suggesting that thermal stress‐induced oxidative damage in poultry may be associated with iron accumulation. When the antioxidant enzyme GPX4 is insufficient to detoxify excessive lipid peroxides, ferroptosis is initiated, resulting in oxidative hepatic injury. Glutathione (GSH) is the essential co‐substrate for GPX4, and its synthesis depends directly on SLC7A11. In addition, the expression of both GPX4 and SLC7A11 is regulated by Sirt1, a critical suppressor of ferroptosis (Chen et al. 2023). In this study, network pharmacology was applied to construct and analyze an interaction network encompassing YOL‐derived components, heat stress‐associated targets, and ferroptosis‐related pathways. Within this network, the deacetylase Sirt1 was identified as a central hub protein. Molecular docking analysis further revealed that active constituents of YOL exhibit favorable binding to the allosteric pocket of Sirt1, suggesting a potential mechanism underlying Sirt1 activation. This in silico prediction was corroborated by experimental data evidence, as YOL treatment significantly upregulated hepatic Sirt1 expression. It's reported that acacetin ameliorates cardiomyopathy via Sirt1‐mediated activation of the Nrf2 signaling pathway (Wu et al. 2020). In this study, heat stress significantly decreased the expression of GPX4 and SLC7A11, indicating that compromised endogenous antioxidant defenses contribute to the facilitation of ferroptotic cell death. Vitamin C partially restored the expression of Sirt1 and GPX4, confirming its antioxidant activity but indicating limited engagement at the signaling pathway level. In contrast, YOL activated both NRF2 and Sirt1 and restored the expression of SLC7A11 and GPX4. These findings suggest that YOL enhances hepatic antioxidant capacity, which is likely associated with the attenuation of ferroptosis.

Mitochondrial morphology is closely associated with the regulation of ferroptosis. The liver is characterized by a high mitochondrial density and intense metabolic activity, rendering it particularly critical for the maintenance of redox homeostasis. In this study, heat stress induced marked mitochondrial ultrastructural damage, including cristae fragmentation and loss, thereby confirming severe mitochondrial injury. Mitophagy serves as a key compensatory mechanism for mitochondrial quality control, in which PINK1 acts as a sensor of mitochondrial damage and Parkin functions as the downstream effector (Narendra and Youle 2024). Previous studies found that mitophagy can be enhanced through the PINK1/Parkin‐dependent pathway, thus alleviating heat stress‐induced cellular damage in broilers (Shi et al. 2024; Ma et al. 2025). Moreover, baicalin, wogonoside, and acacetin have been reported to promote PINK1/Parkin‐dependent mitophagy (Zhou et al. 2025; Wu et al. 2025; Hong et al. 2021). Consistent with the activation of mitochondrial damage signaling, the heat‐stressed group exhibited elevated PINK1 expression accompanied by reduced Parkin levels. YOL restored mitochondrial cristae integrity and increased autolysosome formation, indicating its ability to ameliorate heat stress‐induced mitochondrial structural damage and promote autophagic clearance. Furthermore, YOL reversed the heat stress‐mediated suppression of both PINK1 and Parkin expression. These findings suggest that YOL mitigates oxidative stress and associated liver damage by activating PINK1/Parkin‐dependent mitophagy, thereby improving mitochondrial ultrastructure and facilitating the removal of damaged organelles.

## 5 Conclusion

YOL exerts multi‐target hepatoprotective effects by activating Sirt1, which coordinates KEAP1/NRF2‐mediated antioxidant defense, GPX4/SLC7A11‐dependent ferroptosis inhibition, and PINK1/Parkin‐mediated mitophagy, thereby mitigating heat stress–induced oxidative liver injury.

## Author Contributions

Conceptualization and investigation and funding, Lu‐Ping Tang, Yong‐Ming He. Investigation and methodology and writing – original draft, Zi‐Hao Li and Jia‐Ci Cai. Formal analysis and supervision and software, Yang Yang and Hui‐Lin Li. Data curation and resources, You Li and Shun Tian. All authors have read and agreed to the published version of the manuscript.

## Funding

This project was supported by Guangdong Provincial Department of Science and Technology [Grant no. 2024A1515030170]. The National Natural Science Foundation of China [Grant no. 32402930]. Department of Education of Guangdong Province [Grant no. 2024KTSCX208].

## Ethics Statement

All animals work was conducted in strict compliance with the Guidelines for the Care and Use of Experimental Animals issued by the Ministry of Science and Technology of the People's Republic of China (Approval No. 2006‐398), and approved by the Laboratory Animal Management Committee of Foshan University. All efforts were made to minimize animal suffering.

## Conflicts of Interest

The authors declare no conflicts of interest.

## Data Availability

All data supporting the findings of this study are included in the article. No additional datasets were generated or analyzed.
